# Evaluation of the Ability of Nutri-Score to Discriminate the Nutritional Quality of Prepacked Foods Using a Sale-Weighting Approach

**DOI:** 10.3390/foods10081689

**Published:** 2021-07-22

**Authors:** Edvina Hafner, Igor Pravst

**Affiliations:** 1 Nutrition Institute, Tržaška Cesta 40, SI-1000 Ljubljana, Slovenia; edvina.hafner@nutris.org; 2 Biotechnical Faculty, University of Ljubljana, Jamnikarjeva 101, SI-1000 Ljubljana, Slovenia; 3 VIST–Higher School of Applied Sciences, Gerbičeva Cesta 51A, SI-1000 Ljubljana, Slovenia

**Keywords:** front-of-pack, labelling, Nutri-Score, food supply, nutrient profiling, sale-weighting, Slovenia

## Abstract

The implementation of mandatory front-of-pack nutrition labelling is currently being discussed in the European Union (EU). The Nutri-Score (NS) was developed in France to empower consumers to make informed and healthier food choices. Based on strong evidence of its efficacy in supporting healthy choices, it has already been implemented for voluntary use in some EU member states, making it relevant to developing a harmonised EU scheme. This study aimed to evaluate the NS’s discriminating ability on products available in the food supply and compare it with Slovenian national nutritional recommendations based on an adapted WHO Europe (WHOE) profile. The innovative approach of the study is that we used sale-weighting to address the public health importance of available foods, with consideration of market share. We profiled 15,822 products available in the Slovenian food supply in 2017. The NS had a high ability to discriminate food products based on nutritional composition. Products that are generally encouraged in dietary recommendations (fruits, vegetables, cereals) had, in most cases, better NS grades than less favourable products (confectionery, snack foods, added fats), which is also in line with the national nutrition policy programme. The discriminating ability of the model was also shown within food (sub)categories (e.g., plain and flavoured yoghurt). Sale-weighting showed that offerings do not always reflect sales. Major differences between offerings and sales were observed for beverages, dairy, fruits/vegetables, and edible oils/emulsions. Additionally, sale-weighted distribution tended towards less favourable nutritional composition, particularly in categories with overall smaller offerings of products with favourable composition. The NS showed moderate agreement with the WHOE profile (κ = 0.57); differences were particularly observed in flavoured yoghurts, juices, cooking oils, and cheeses. Modelling the operation of the NS with representative real-life food samples provided insight valuable for developing and implementing harmonised mandatory front-of-pack nutrition labelling in Europe.

## 1. Introduction

Noncommunicable chronic diseases such as diabetes, cardiovascular diseases, and cancer are the leading causes of death, responsible for over 70% of deaths worldwide, with Europe being one of the most affected regions [1,2]. An unhealthy diet is considered to be one of the key preventable risk factors in these diseases. Providing information through food labels has been shown to be an important driver stimulating consumers to make healthier food choices [3]. Information about the nutritional composition of foods is typically provided on back-of-package labels (BOPLs), usually in tables with numeric information about the contents of energy and specific nutrients. Studies have shown that nutritional information on BOPLs is useful for consumers; however, they are not very effective at stimulating healthier food choices and improving overall diet quality [4] since they are often overlooked and misinterpreted due to lack of knowledge and motivation [5].

Therefore, in recent years, there has been a greater emphasis on front-of-pack nutrition labelling (FOPNL) to provide relevant nutrition information in a convenient and understandable way. Such labelling could assist all consumers (regardless of their nutrition knowledge) with making not only informed but also healthier food choices. FOPNL also has a vital role in encouraging the food industry to launch food products with improved nutritional composition [6]. The European Commission has also recognized this and proposed the introduction of a mandatory harmonised FOPNL as part of the Farm to Fork Strategy. A proposal for such a labelling scheme is expected by the end of 2022 [7].

The Nutri-Score (NS) is an example of an FOPNL scheme that is currently in use in some EU member states on a voluntary basis. The NS is a summary FOPNL that grades the nutritional quality of products with a five-colour scale. Products are sorted into groups based on the United Kingdom Food Standards Agency (FSA) nutrient profiling system (NPS), which aims to limit children’s exposure to less healthy foods through television advertising [8]. The FSA-NPS score is composed of positive and negative points based on the nutritional composition of 100 g or 100 mL of product. This system ends up with a binary result, which can promote dichotomous thinking, prompting the idea that products are either “good” or “bad” [9,10]. With that in mind, the French High Council for Public Health (HCSP, Haut Conseil de la Santé Publique) adjusted this concept and named it FSAm-NPS. Further, they created FOPNL with multiple categories, with grades from dark green (most healthy) to red (least healthy) [11]. Letters were added to colours for simplification purposes, primarily for colour-blind people [12]. The French Agency for Food, Environmental, and Occupational Health and Safety (Agence nationale de sécurité sanitaire de l’alimentation, de l’environnement et du travail—ANSES) highlighted that the algorithm had some limitations in certain food groups, showing inconsistencies with nutritional recommendations [13], which resulted in its being adapted for cheeses, added fats, and beverages [14]. FOPNL is now also used and endorsed by the authorities of six other European countries (Germany, Belgium, Luxembourg, Netherlands, Switzerland, and Spain) [15].

Several studies have investigated the effectiveness of the NS to support healthier food choices. The first studies were conducted in France before and during the implementation period, followed by mostly supportive evidence from other countries. In various settings, the NS has shown to be a very effective FOPNL for identifying healthier products [16,17], improving people’s food choices [18,19] and purchases [20,21]. The NS is also well perceived by consumers, ranking high with regard to liking and understanding [22,23]. Recent results from modelling and cohort studies also suggest an association between consumption of products with poor NS grades and a higher risk for noncommunicable chronic diseases and even mortality, indicating the high public health potential of this scheme [24,25,26]. The NS is strongly supported by scientists across Europe [27]; some EU member states and some major food companies have already adopted it. However, there is also some opposition to the NS, mostly by food companies and trade associations concerned about the possible effects of implementing the scheme on foods high in nutrients of concern. Some EU member states have expressed concern that such a scheme would jeopardize their traditional diets [15,28]. While a large body of evidence supports the efficacy of the NS, most of the data were collected in Western Europe. To support further EU-wide food policy decisions considering the diversity of European food cultures and habits, studies from other EU regions are needed [15].

Discriminating ability and consistency with dietary recommendations are two key aspects of validating a nutrient profile model [29,30]. While some previous studies have tested the discriminating ability of the NS on large food supply samples [9,12,31,32,33], they have treated all available foods equally, regardless of their market share. Actually, the lack of analysis based on real-life retail data is considered an important knowledge gap in the area of food labelling [34]. In addressing this challenge, sale-weighting has been shown to be valuable in supporting the monitoring of changes in food supply and estimating the content of critical nutrients, such as sugar [35] and salt [36]. While sale-weighting the food supply would provide a more realistic perspective, in which consumers would be exposed to products with different NS grades, such an approach has never been used. It should also be noted that in existing studies, the consistency of the NS with dietary recommendations has commonly only been described, without deeper insights into specific food categories.

Considering the challenges mentioned above, our aim was to evaluate the ability of the NS to discriminate the nutritional quality of prepacked products using a case study with a cross-sectional, nationally representative Slovenian food supply dataset, together with national 12-month sales data. Considering that there are no data available on the consistency of the NS with Slovenian national dietary recommendations across a variety of food categories, two approaches were used to investigate this. First, we compared NS grades with category-based dietary guidelines provided in the national nutrition policy programme (National Program on Nutrition and Health Enhancing Physical Activity 2015–2025 [37]). This programme specifies dietary goals and recommendations for different age groups. The second approach was to investigate the agreement between the NS and the nationally adapted WHO Europe (WHOE) profile [38], which is used to restrict the advertising of unhealthy foods to children.

## 2. Materials and Methods

### 2.1. Data Collection

Cross-sectional data on available foods and their composition were retrieved from the Composition and Labelling Information System (CLAS) database (Nutrition Institute, Ljubljana, Slovenia), which collects information on prepacked products in the Slovenian food supply [39]. The methodology for data collection is described in detail elsewhere [40]. In brief, CLAS ensures a nationally representative sample for monitoring studies in grocery stores from retailers that represent the majority of the national food market share. We used available food supply data collected in 2017 in two megamarkets (Mercator Center, Interspar), two supermarkets (Tuš, Spar), and two discount markets (Lidl, Hofer). All prepacked food products with unique European Article Number (EAN) barcodes were systematically photographed and collected in the CLAS database. Information about the nutritional compositions of foods and ingredients was extracted from the photographs.

Sales data were obtained from retailers. We were able to obtain sales data from two retailers, which represent the majority of the national market. These data referred to national 12-month (2017) sales of specific products. We included sales data for products that were part of the food composition database mentioned above. Information on sales is given in a universal form, which includes EAN barcode, product name, number of products sold per year, and package quantity (kg or L). The two datasets were connected with matching EAN barcodes. We should note that we had access to retail sales data for over 50% of the national food supply; some retailers were unwilling to disclose this information.

### 2.2. Food Categorization and Exclusion Criteria

Food products were categorised according to the classification proposed by Dunford et al. [41]. Minor modifications were made due to specific aspects of the European market and particular objectives of the study. We excluded foods in categories that are part of Appendix V of Regulation No. 1169/2011 [42] and do not have mandatory nutritional declarations (*n* = 3107). According to the latest Santé Publique France recommendations for NS profiling [14], we further excluded the categories of baby foods and meal replacements for weight loss (*n* = 88). Individual products were excluded if they were missing part of the mandatory nutritional declaration that was needed for NS calculation (*n* = 1414) [42] or were prepared with the addition of another ingredient (*n* = 484). In order to avoid possible errors in the labelled nutrition declarations, we also excluded products for which the deviation between declared and calculated energy values was more than 20% (*n* = 175). The calculation of energy value for this check was conducted with the use of conversion factors, provided in Regulation No. 1169/2011 [42].

The starting dataset contained a total of 21,090 products. After considering the exclusion criteria, we ended up with 15,822 products, which were classified into 13 main categories and 56 subcategories.

### 2.3. Nutri-Score Calculation

To determine NS grade, we first calculated the FSAm-NPS score. For this, we needed the nutritional composition of 100 g of food (or 100 mL of beverage). Positive points (0–10) were assigned to negative attributes of food, including energy (kJ), total sugars (g), saturated fatty acids (SFAs) (g), and sodium (mg), while negative points (0–15) represented positive attributes, such as fibre (g), proteins (g), and percentage of fruits/vegetables/pulses/nuts/specific oils (FVPN %). The sum of all positive points (0 to 40) and all negative points (−15 to 0) gave the product’s final score [13]. After we calculated the FSAm-NPS score, we assigned NS grades: A, −1 and below (dark green); B, 0 to 2 (light green); C, 3 to 10 (yellow); D, 11 to 18 (orange); and E, 19 and above (red). For beverages, grades were slightly modified: A, only water (dark green); B, up to 1 (light green); C, 2 to 5 (yellow); D, 6 to 9 (orange); and E, 10 and above (red). Details on the NS scoring system and specifics of beverages, cheeses, and added fats are described elsewhere [9,11,13].

Some information, such as fibre and FVPN %, is not part of the mandatory food labelling. Therefore, we supplemented missing data using all relevant information available for specific products. When the mean amount of dietary fibre in a whole subcategory was below 0.9 g (not relevant for NS), we assigned a value of 0 to all products with the missing data in the subcategory. This was the case for the following subcategories: canned fish and seafood, cheese and processed cheese, cooking oils, cream, flavoured yoghurt, ice cream and edible ices, jelly, juices, mayonnaise and dressings, milk and milk drinks, nectars, plain yoghurt, processed meat, soft drinks, unprocessed fish, unprocessed meat, and yoghurt imitates. For other subcategories, we calculated missing data for dietary fibre from energy value according to the formula energy (kJ) = total carbohydrates (g) × 17 + total protein (g) × 17 + total fat (g) × 38 + alcohol (g) × 29 + total dietary fibre (g) × 8. To avoid possible errors, we individually checked all products for which the final NS grade was affected by the calculated amount of dietary fibre. Where the calculated amount was not realistic (exceeding 2SD for this subcategory), the amount of dietary fibre was estimated by manual matching with similar products using a previously described approach [43]. FVPN % was assessed based on the ingredients list. When the list did not provide sufficient information to estimate FVPN %, we referred to legislation to determine the minimum amount of FVPN for some categories (e.g., jams and nectars) [44,45]. For dried fruit, vegetables/pulses, and concentrated vegetables/pulses, FVPN % was multiplied by a factor of 2, per recommendations [14].

### 2.4. Statistical Analyses

Statistical analyses were performed using Microsoft Excel 2019 (Microsoft, Redmond, WA, USA) and R: A Language and Environment for Statistical Computing 2020 (R Core Team, Vienna, Austria).

We assessed the distribution of prepacked products across different NS grades for main categories and subcategories and displayed this information in boxplots emphasizing median, 25th, and 75th percentiles. Discriminating ability was considered good when the food group comprised at least three different NS grades [31].

To assess consistency with nutritional recommendations, we compared the results with the national nutrition policy programme [37] and calculated the agreement between the NS and the nationally adapted WHOE profile [38] using Cohen’s kappa. For each profile, the proportion of “healthy” products was calculated based on the following criteria: WHOE: permitted for marketing to children; NS: A (dark green) or B (light green) [46].

For sale-weighting, we calculated the total amount (kg or L) of products sold per year using the number of products and package quantity. We displayed the corrected distribution and compared it with the offerings (products available at the time of sampling).

## 3. Results

### 3.1. Distribution and Discriminating Ability

**Figure 1:**
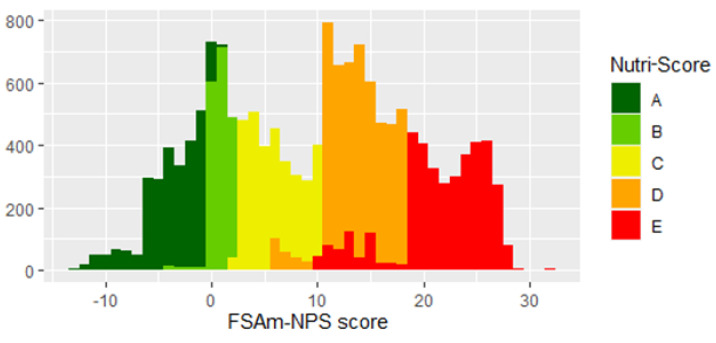
Overall distribution of FSAm-NPS score across the Slovenian food supply (*n* = 15,822). Letters from A to E represent five Nutri-Score grades.

Our final sample consisted of 15,822 products, including 2578 dairy products, 1999 bread and bakery products, 1938 confectionery products, 1822 fruits and vegetables, 1644 meat and meat products, 1636 cereal and cereal products, 1152 beverages, 973 sauces and spreads, 571 convenience foods, 503 fish and fish products, 492 edible oils and emulsions, 485 snack foods, and 29 types of eggs. The overall distribution of products across NS grades is displayed in Figure 1. Overall, 16.5% of products were classified as grade A, 11.5% as B, 18.6% as C, 29.2% as D, and 24.2% as E. The mean FSAm-NPS score was 9.9 ± 9.5 points. This distribution also shows spikes, indicating that several products are on the border of higher NS grades (*n* = 2378; 15%).

The ability of the NS to discriminate between different products was good in most food categories. Actually, 12 out of 13 main categories and 83.9% of subcategories (*n* = 47) contained products classified with three or more NS grades. The categories where NS was not able to discriminate between products (only 1 or 2 NS grades) were homogeneous subcategories (e.g., water, eggs) and subcategories with a small number of products (e.g., cheeses, coffee mixes) (see Table S1).

**Figure 2:**
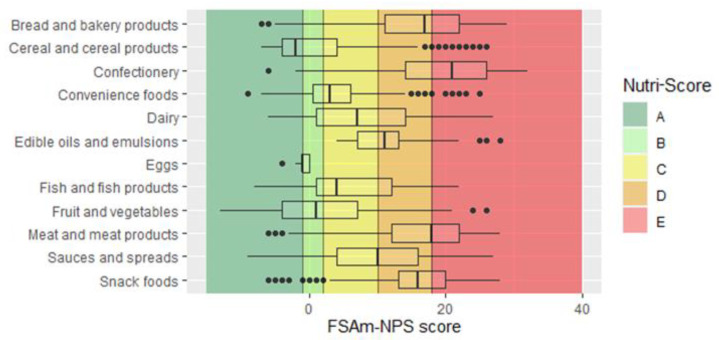
Distribution of FSAm-NPS score for main food categories (*n* = 15,822). Vertical lines represent borders of five Nutri-Score grades from A to E. Left boundary of the box represents 25th percentile, line in the box is median, and right boundary represents 75th percentile. Error bars represent lower limit (25th percentile − 1.5 × (interquartile range)) and upper limit (75th percentile + 1.5 × (interquartile range)). Black circles are individual outliers.

The NS was able to discriminate between main categories in line with national dietary recommendations (Figure 2). Food categories that should be limited according to dietary recommendations, such as sweets, fats, and snack foods, had lower NS grades (D or E) on average compared with categories whose consumption is encouraged, such as fruits, vegetables, and cereals (mainly A and B). The large variability within some categories indicates group heterogeneity and the NS’s ability to discriminate between foods, which is clearly seen if we delve into each category.

**Figure 3:**
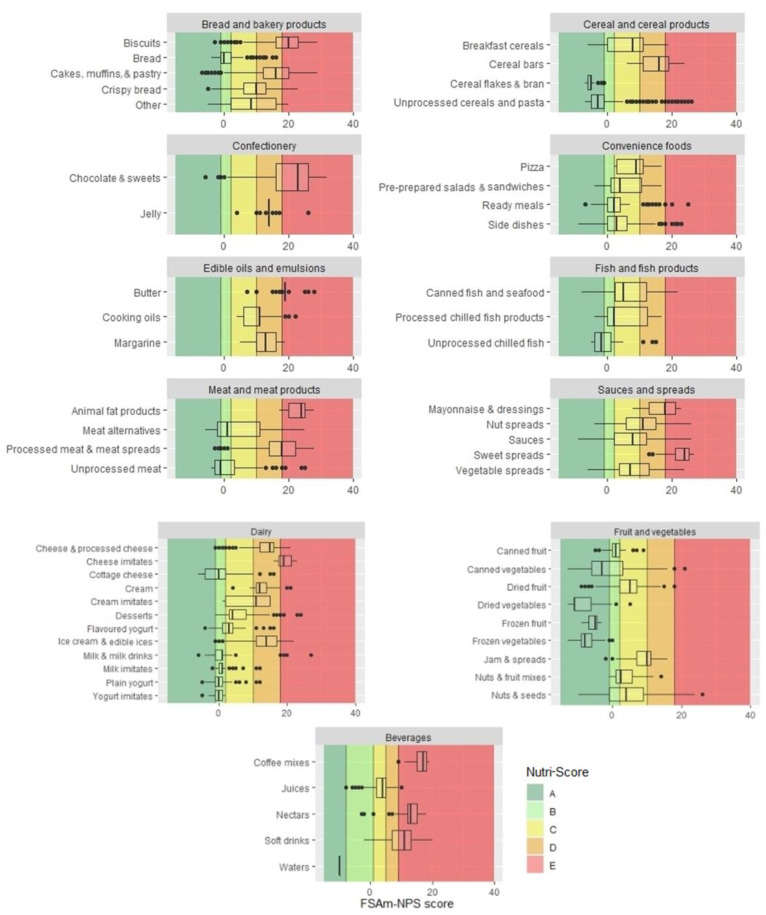
Distribution of FSAm-NPS score for subcategories (*n* = 15,822). Vertical lines represent borders of five Nutri-Score grades from A to E. Left boundary of the box represents 25th percentile, line in the box is median, and right boundary represents 75th percentile. Error bars represent lower limit (25th percentile − 1.5 × (interquartile range)) and upper limit (75th percentile + 1.5 × (interquartile range)). Black circles are individual outliers.

The NS model proved to have good discriminating ability, particularly between subcategories in the same main category (Figure 3). Among beverages, all waters were graded A, while the main grades for juices and soft drinks were C (55.2%) and E (64.2%). A similar ability was shown in distinguishing the nutritional composition of yoghurts and added fats. Plain yoghurts mostly had a grade of B (53.5%), while flavoured yoghurts usually had a C (51.4%). The typical grade of cooking oils was C (49.6%), and that of butter was mostly E (76.7%). The NS could also differentiate foods by processing. Unprocessed cereals had in most cases a grade of A (75.3%), while more processed breakfast cereals mostly had C (42%). Similar differences were seen with fruit: frozen fruit was graded A (100%), canned fruit mostly B (77.2%), and dried fruit C (57%) (see Table S1). Similar results were seen for unprocessed meat and fish, which had better grades (mostly A) than processed alternatives, which had average grades of D or E. For bread and bakery products, we noted a difference between whole grain and refined products, with whole grains typically rated one grade better. Overall, recommended foods mostly had better grades than less desirable alternatives.

The NS was consistent with the national nutrition policy programme, which aims to increase the consumption of fruits and vegetables and reduce the consumption of sugary drinks, confectioneries, and other sweets. The programme also promotes reduced salt and moderate fat intake. Among more favourable foods are those with higher content of unsaturated fats (such as olive and rapeseed oil) and reduced fat content (such as skim milk products). The programme favours the consumption of meat with visible fat removed and, in general, discourages excess consumption of meat products. It also promotes the consumption of fibre-rich foods, with an emphasis on whole grains [37].

### 3.2. Consideration of Market Shares and Sale-Weighting

**Figure 4:**
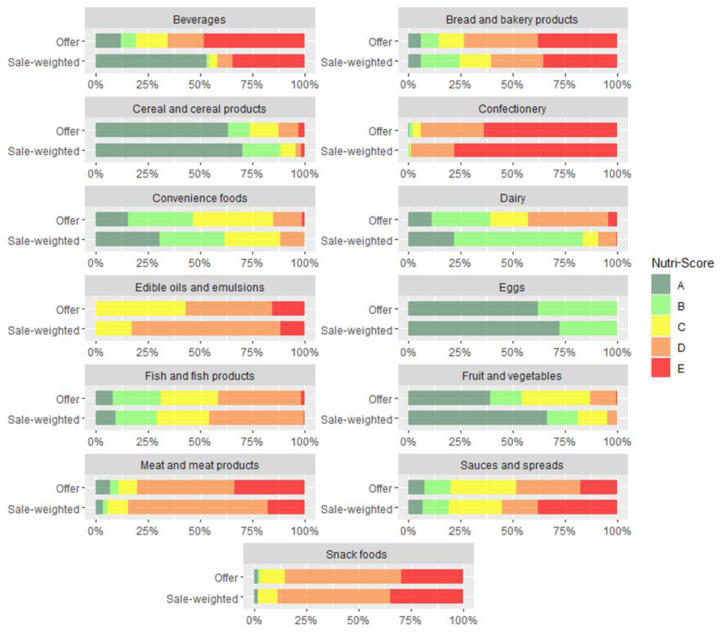
Distribution of Nutri-Score grades across main categories using sale-weighting (*n* = 10,402). Letters from A to E represent five Nutri-Score grades.

To investigate to what extent consumers would be exposed to different NS grades in specific food groups, we applied the sale-weighted approach. The analysis was performed on a subsample of foods for which 12-month national sales data were available (*n* = 10,402). The results show that in most categories, food supply approximately reflected sales data, but larger differences were observed in specific categories. In seven categories, sale-weighted data showed more favourable distribution of NS, with a higher proportion of better NS grades (Figure 4). This difference was particularly obvious among beverages, where waters (graded A) represented more than half (53.1%) of all sold beverages. A notable difference was also seen for dairy products, among which healthier products (graded A or B) represent 39.1% of offerings and 83.4% of sales. A similar result was seen for fruits and vegetables, with 54.1% of offerings of healthy products but 80.9% of sales. The sale of products with lower nutritional quality (graded D or E) was higher for six categories: edible oils and emulsions, confectionery, snack foods, sauces and spreads, fish and fish products, and meat and meat products. Most of these categories also had a lower proportion of healthy offerings. Offerings were not aligned with sales data, especially for edible oils and emulsions, where proportions of offerings with NS grades of C (43.1%) and D (41.5%) were similar, but after sale-weighting, products graded D represented 70.9% of sales. A difference was also seen for sauces and spreads, where products with the lowest nutritional quality (graded E) represented only 17.7% of offerings but 38.1% of sales (see Table S2).

### 3.3. Agreement between Nutri-Score and Nationally Adapted WHO Europe Nutrient Profile

Overall, the NS showed moderate agreement with the nationally adapted WHOE profile, with κ = 0.57 (Table 1). The agreement was almost perfect for cereals and cereal products (κ = 0.84), and substantial for crispy bread (κ = 0.70), unprocessed cereals and pasta (κ = 0.77), side dishes (κ = 0.64), milk and milk drinks (κ = 0.62), processed chilled fish products (κ = 0.79), and snack foods (κ = 0.69). Lower agreement was noted for flavoured yoghurt (κ = 0.06), cheese and processed cheese (κ = 0.04), milk imitates (κ = 0.08), soft drinks (κ = 0.09), and dried fruit (κ = 0.10). Cohen’s kappa cannot be computed if one or both profiles include or exclude the whole category. Therefore, we also calculated % of agreement, which was perfect (100%) for 12 subcategories and lower for juices (20%), cooking oils (9.1%), canned fruit (8.7%), and yoghurt (6.1%) (see Table S1). Overall, the WHOE profile was shown to be stricter, especially for fruits and vegetables, dairy, and sauces. The NS was stricter for meat and meat products, beverages, edible oils, and emulsions.

## 4. Discussion

This study shows that the NS has a good ability to discriminate among food products not only between but also within categories. This ability is aligned with the national nutrition policy programme [37] and has moderate agreement with the nationally accepted WHOE profile [38].

The ability to discriminate among products was high for the vast majority of the main categories (11 out of 12) and subcategories (83.9%). The NS could discriminate products within and across categories and subcategories with at least three different NS groups. The categories where it was not able to discriminate were categories with very similar products and small numbers of items. These results are consistent with previous studies conducted on the French [9] and German [12] food supplies and with a comparative study that included eight European countries, showing a discriminating ability between 70% and 88% [31]. Reports based on the Open Food Facts collaborative project in 13 European countries also support these results [32,33]. Our results, therefore, additionally validate the NS’s discriminating ability, which is a significant supportive element of FOPNL that can help guide consumers [12] and is a key step in validating nutrient profiles [29].

The high variability within categories (especially in those with five different NS grades) can also encourage food manufacturers to use the NS as a competitive tool and consequently improve the nutritional composition of their products [9]. In fact, the food reformulation aspect is one of the most important attributes of FOPNL [6,34]. For this purpose, the NS was built based on the distribution of products in the food supply [10]. This aim is also seen in overall distribution, shown in our study with the food supply in Slovenia, although the NS was originally developed in France. Looking at the overall distribution of products in Slovenia, we can see that there are spikes at the borders, indicating better NS grades (Figure 1). These spikes represent about 15% of all products in the Slovenian food supply. A similar distribution is seen in other European countries [12,32,33]. This indicates that the implementation of the NS could encourage many producers to make minimal but important improvements to these products in order to get better NS grades. This would be beneficial for public health and would also give producers realistic goals for food reformulation [47]. Together with other studies that show the ability of the NS to affect consumer purchasing behaviours [20,21], these results are promising, indicating that it could also encourage consumers to choose reformulated products, which is important information for the food industry. We should also note that the observed average FSAm-NPS score in Slovenia is higher (9.9, indicating a higher proportion of less healthy foods) compared with those in previously investigated European countries (7.1–9.6) [33], with a large proportion of products graded D (24.2%). The reformulation aspect of FOPNL is therefore even more important regionally, and should be encouraged by policymakers. Supporting the provision of reformulated foods is also an important part of the national nutrition policy programme [37].

Compliance with local dietary recommendations is another essential part of FOPNL assessment [48]. In most cases, the NS was aligned with Slovenian nutritional recommendations. More than half of the products in the fruits, vegetables, and cereals categories, consumption of which is encouraged, were graded A or B, while less desirable categories, such as confectioneries and snack foods, were mostly graded D or E. As mentioned, the high variability within each category allows consumers to differentiate between similar products and choose the healthiest [29]. This ability was evident for products in the same main category, where favourable products, such as plain yoghurt, unprocessed cereals, and cooking oils, had better grades than less desirable alternatives, such as flavoured yoghurt, breakfast cereals, and butter. For bread and bakery products, the NS indicated a preference for whole grain over refined products. Fruits generally had good grades, but the system still distinguished between less and more appropriate products, giving priority to frozen fruit (graded A), followed by canned fruit (B) and dried fruit (C). Among beverages, water was graded A, juices mostly C, and soft drinks mostly E, which is consistent with nutritional recommendations. In general, the NS rated products better if they were less processed and lower in salt, sugar, and fat. All of these results align with the national nutrition policy programme, which aims to increase the consumption of fruits, vegetables, and whole grains; limit fats; and reduce the intake of sweet and salty foods [37]. Similar results were found when the NS was compared with nutritional recommendations in other European countries [12,31,32,33].

To get further insights, we also weighted food offerings with sales data. The results show that offerings often reflect sales, but this is not always the case. For seven categories, the NS indicated that sales distribution was more favourable (with a higher proportion of better grades) than offerings, with notable differences in beverages, dairy, fruits, and vegetables. Meanwhile, sales distribution was less favourable than offerings in six categories, mainly those in which the offering of healthy products is limited (confectionery, snack foods, meat and meat products, fish and fish products, sauces, edible oils, and emulsions). These are food categories where the use of FOPNL would be particularly helpful for consumers to find and choose healthier products. Additionally, encouraging healthier choices and reformulating products with the lowest nutritional quality were shown to be particularly beneficial for public health [49]. Studies that include real-life retail data are rare and generally represent a major knowledge gap in different FOPNL schemes [34].

To further investigate the compliance of the NS with national dietary recommendations, we compared the NS profile with the nationally adapted WHOE profile. It is important to point out that, in principle, the WHOE is a binary profile that can have only two possible results—permitted for marketing or not [38]—making it challenging to compare it with scoring systems, such as the NS [10]. To enable comparison, we considered NS grades A and B as passing. Considering this, the overall agreement between the NS and the WHOE was moderate (κ = 0.57), with an almost perfect agreement for cereals (κ = 0.84) and a perfect agreement (100%) in 12 subcategories. Although the overall % of agreement was high (82.9%), differences were noted for some important food categories, such as flavoured yoghurts, juices, cooking oils, and cheeses. This can be explained by differences in the nutrient profiling in the two models. In the NS, all foods are subjected to scoring, while the WHOE, in some cases, includes or excludes an entire food category (e.g., juices or cooking oils). Additionally, the WHOE profile excludes some products based on added sugars, while the NS takes into account the amount of total sugars. This makes the WHOE profile stricter for many products, particularly dairy and processed fruit. However, the difference in the algorithms of the two models could also be considered a benefit of the NS, to be able to differentiate and support reformulation. While in some categories food manufacturers cannot achieve any improvement in the WHOE profile, the NS enables them to make small changes in the food composition to get better grades.

The reported differences in nutrient profiling approaches of different labelling schemes highlight areas that need to be carefully addressed during the harmonisation of FOPNL in Europe. Consistency among labelling profiles is vital in order to provide unambiguous information to consumers [50,51]. For example, the NS for fats and cheeses is mentioned as being of concern in some countries, and some countries point out inconsistencies with their national recommendations [28,52]. Since only a few products in these categories can have a grade of A or B (e.g., cottage cheese), they mention the risk that consumers would interpret such food groups as unhealthy. Considering that high-fat products should be consumed in moderation, it is quite logical that their grades are not as high as those of fruits and vegetables, for example, but it is an important feature of the NS that product differentiation is assured in these categories. For example, among cheeses, the NS encourages consumption of low-fat products, while among vegetable oils, it penalises those with high saturated fat content (e.g., coconut and palm oil) and highlights those with favourable nutritional composition, such as olive oil. This approach makes the NS closely align with dietary guidelines in many countries [53,54]. However, these results indicate the importance of clear communication and interpretation of grading-type FOPNL schemes for consumers in order to avoid misinterpretation. This way, food labelling schemes can guide consumers towards healthier choices, rather than give conflicting information [10].

The main advantage of this study is the use of a nationally representative cross-sectional sample of prepacked foods (*n* = 15,822); additionally, we were able to account for market share differences using a sale-weighting approach. Major national retailers provided 12-month sales data for individual foods, which facilitated insights into the market situation and can support monitoring changes in the food supply in the future.

Some study limitations also need to be mentioned. We were unable to obtain sales data for some retailers. However, sales data were available for retailers covering more than 50% of the market share. In our dataset, for example, sales data were available for 63% of foods, enabling us to use the sale-weighting approach on a very large database (*n* = 10,402). To exclude possible methodological errors related to missing sales data, the two datasets were carefully compared, and the distribution of NS grades across products included in sale-weighting (*n* = 10,402) was very similar to the whole sample (*n* = 15,822) (Table S2). Previous studies examining the discriminating ability of the NS used Open Food Facts [12,32,33] and EuroFIR [31] datasets, with different sampling approaches. A direct comparison of results between studies is also challenging because of differences in the food categorisation systems used. To ensure international comparability in future studies, the results reported here are presented using food categorisation proposed by the Global Food Monitoring Group [41]. We should also mention another limitation, that mandatory food labelling does not always provide all the information needed for the computation of NS scores; therefore, for some foods, missing data (dietary fibre content and % FVPN) were estimated with the use of regulated procedures [44,45] and previously developed methods [31,43]. Additionally, the indicator for assessing discriminating ability was not unified; we selected a method that had already been used in previous studies [12,32,33].

## 5. Conclusions

We showed that the NS can efficiently discriminate among food products both between and within food categories, which can help guide consumers towards healthier food choices. We also showed that the NS is aligned with national dietary recommendations, and that it is in moderate agreement with the nationally adapted WHOE profile. Some differences were observed, particularly among flavoured yoghurts, juices, cooking oils, and cheeses. Applying the sale-weighing approach showed that the distribution of offerings sometimes does not reflect sales. Major differences were observed in four categories: beverages, dairy, fruits/vegetables, and edible oils/emulsions. After correcting for market shares, the distribution of NS grades became less favourable (with a higher proportion of less healthy foods) in six main categories. In most cases, these were categories with overall low availability of healthy products (grade A or B). The results of this study are consistent with those of previous studies that examined the NS’s discriminating ability and consistency with dietary recommendations, and add new insights considering very different market shares of products that are available in the food supply. While the NS was easy to use and performed very well, we also identified some challenges that should be addressed during the implementation of such a system in new markets. To avoid potential misinterpretation, it would be useful to synchronise the introduction of such a scheme with an educational campaign to inform consumers about how to correctly interpret the information on food labels. The findings of this study can support policymakers in making evidence-based decisions about the implementation of harmonised FOPNL in Europe.

## Figures and Tables

**Table 1:** Agreement between the Nutri-Score and the nationally adapted WHOE profile for main categories using Cohen’s kappa and percentage of agreement.

Category	Cohen’s Kappa	% of Agreement
Beverages (*n* = 1152)	0.49	79.5
Bread and bakery products (*n* = 1999)	0.57	91.5
Cereal and cereal products (*n* = 1636)	0.84	93.2
Confectionery (*n* = 1938)	Na	98.2
Convenience foods (*n* = 571)	0.59	80.6
Dairy (*n* = 2578)	0.39	74.9
Edible oils and emulsions (*n* = 492)	Na	26
Eggs (*n* = 29)	Na	100
Fish and fish products (*n* = 503)	0.57	77.7
Fruit and vegetables (*n* = 1822)	0.42	70
Meat and meat products (*n* = 1644)	0.54	89.1
Snack foods (*n* = 485)	0.69	98.6
Sauces and spreads (*n* = 973)	Na	80.7
Total (*n* = 15,822)	0.57	82.9

## Supplementary Materials

The following are available online at https://www.mdpi.com/article/10.3390/foods10081689/s1, Table S1: Discriminating performance of Nutri-Score and agreement between Nutri-Score and adapted WHO Europe profile. Table S2: Distribution of NSC grades in the Slovenian food supply.
