# Supplementary material for: Evaluation of the Ability of Nutri-Score to Discriminate the Nutritional Quality of Prepacked Foods Using a Sale-Weighting Approach
Source: Foods. 2021 Jul 22;10(8):1689. doi: 10.3390/foods10081689 (PMC8392670; doi:10.3390/foods10081689)
Supplement: Supplementary file 1 [file foods-10-01689-s001.zip › foods-1276808-supplementary.pdf]

Supplementary Table S1. Discriminating performance of Nutri-Score and agreement between Nutri-Score and adapted WHO Europe profile (N = 15,822).

Category	Nutri-Score		Nutri-Score: WHO Europe	
	N[a]	MG[b]	Cohen's kappa	% of agreement
**Beverages (n = 1152)**	**5**	**E (47.1%)**	**0.49**	**79.5**
Juices (n = 250)	4	C (55.2%)	na	20
Nectars (n = 139)	3	E (84.9%)	0.43	96.4
Soft drinks (n = 598)	4	E (64.2%)	0.09	94.8
Coffee mixes (n = 33)	2	E (97%)	na	100
Waters (n = 132)	1	A (100%)	na	100
**Bread and bakery products (n = 1999)**	**5**	**E (40.1%)**	**0.57**	**91.5**
Bread (n = 190)	4	B (44.7%)	0.33	66.3
Crispy bread (n = 198)	5	D (44.9%)	0.7	89.9
Other (n = 84)	5	D (35.7%)	0.57	82.1
Biscuits (n = 1,012)	5	E (60.8%)	na	97.4
Cakes, muffins and pastry (n = 515)	5	D (49.1%)	na	91.5
**Cereal and cereal products (n = 1636)**	**5**	**A (61.2%)**	**0.84**	**93.2**
Cereal bars (n = 69)	3	D (42%)	na	100
Breakfast cereals (n = 345)	5	C (42%)	0.59	85.2
Cereal flakes and bran (n = 31)	1	A (100%)	na	96.8
Unprocessed cereals and pasta (n = 1191)	5	A (75.3%)	0.77	95
**Confectionery (n = 1,938)**	**5**	**E (62.7%)**	**na**	**98.2**
Chocolate and sweets (n = 1,758)	5	E (69.1%)	na	98
Jelly (n = 180)	3	D (96.1%)	na	100
**Convenience foods (n = 571)**	**5**	**C (38.2%)**	**0.59**	**80.6**
Pizza (n = 52)	3	C (59.6%)	0.24	90.4
Ready meals (n = 264)	5	B (38.6%)	0.56	77.7
Pre-prepared salads and sandwiches (n = 83)	4	C (33.7%)	0.54	79.5
Side dishes (n = 172)	5	C (35.5%)	0.64	82.6
**Dairy (n = 2578)**	**5**	**D (38.3%)**	**0.39**	**74.9**
Cottage cheese (n = 76)	4	A (47.4%)	0.23	80.3
Cheese and processed cheese (n = 720)	5	D (79%)	0.04	75.8
Cheese imitates (n = 6)	2	E (66.7%)	na	100
Plain yoghurt (n = 226)	4	B (53.5%)	0.38	80.5
Flavoured yoghurt (n = 531)	4	C (51.4%)	0.06	55.9
Yoghurt imitates (n = 33)	2	B (57.6%)	na	6.1
Milk and milk drinks (n = 172)	5	B (54.7%)	0.62	88.2
Milk imitates (n = 149)	4	B (67.8%)	0.08	36.2
Cream (n = 124)	3	D (78.2%)	na	100
Cream imitates (n = 27)	3	D (55.6%)	na	66.7
Desserts (n = 121)	5	C (62%)	na	83.9
Ice cream and edible ices (n = 393)	5	D (64.4%)	na	98.7
**Edible oils and emulsions (n = 492)**	**3**	**D (42.9%)**	**na**	**26**
Butter (n = 60)	3	E (76.7%)	na	100
Margarine (n = 61)	3	D (68.9%)	na	55.7
Cooking oils (n = 371)	3	C (49.6%)	na	9.2
**Eggs (n = 29)**	**2**	**A (62.1%)**	**na**	**100**
**Fish and fish products (n = 503)**	**5**	**D (32.8%)**	**0.57**	**77.7**
Canned fish and seafood (n = 358)	5	D (38.5%)	0.46	73.2
Unprocessed chilled fish (n = 77)	4	A (64.9%)	na	88.3
Processed chilled fish products (n = 68)	4	B (38.2%)	0.79	89.7
**Fruit and vegetables (n = 1822)**	**5**	**A (39.9%)**	**0.42**	**70**

Category	Nutri-Score		Nutri-Score: WHO Europe	
	N[a]	MG[b]	Cohen's kappa	% of agreement
Frozen vegetables (n = 127)	2	A (99.2%)	na	100
Canned vegetables (n = 620)	5	A (64.4%)	0.33	62.6
Dried vegetables (n = 46)	3	A (87%)	0.19	73.9
Frozen fruit (n = 31)	1	A (100%)	na	100
Canned fruit (n = 92)	3	B (77.2%)	na	8.7
Dried fruit (n = 221)	4	C (57%)	0.1	68.8
Nuts and fruit mixes (n = 50)	4	B (46%)	0.24	62
Jam and spreads (n = 296)	4	C (52.4%)	0.51	96.3
Nuts and seeds (n = 339)	5	C (38.9%)	0.34	64.9
**Meat and meat products (n = 1644)**	**5**	**E (41.7%)**	**0.54**	**89.1**
Unprocessed meat (n = 115)	5	A (50.4%)	na	73
Processed meat and meat spreads (n = 1364)	5	D (45.7%)	0.27	91.5
Animal fat products (n = 61)	2	E (95.1%)	na	95.1
Meat alternatives (n = 104)	5	A (42.3%)	0.45	71.2
**Snack foods (n = 485)**	**5**	**D (56.7%)**	**0.69**	**98.6**
**Sauces and spreads (n = 973)**	**5**	**C (33.8%)**	**na**	**80.7**
Sauces (n = 645)	5	C (40.6%)	na	74.9
Mayonnaise and dressings (n = 104)	3	E (48.1%)	na	100
Sweet spreads (n = 80)	2	E (87.5%)	na	100
Nut spreads (n = 44)	5	D (40.9%)	na	88.6
Vegetable spreads (n = 100)	5	C (44%)	na	79

[a] Number of different Nutri-Score grades in main category and subcategories.
[b] Main grade (MG): predominant Nutri-Score grade in main category and subcategories.
na, not applicable.

Supplementary Table S2. Distribution of Nutri-Score grades in Slovenian food supply.

Category	Offerings full dataset (N = 15,822)					Offerings foods with sales data (N = 10,402)					Sale-weighted offerings foods with sales data (N = 10,402)				
	**A (n (%))**	**B (n (%)))**	**C (n (%))**	**D (n (%))**	**E (n (%))**	**A (n (%))**	**B (n (%))**	**C (n (%))**	**D (n (%))**	**E (n (%))**	**A (%)**	**B (%)**	**C (%)**	**D (%)**	**E (%)**
Beverages	132 (11,5%)	78 (6,8%)	176 (15,3%)	223 (19,4%)	543 (47,1%)	100 (12%)	60 (7.2%)	125 (15.1%)	143 (17.2%)	402 (48.4%)	53.1	1.3	3.6	7.6	34.5
Bread and bakery products	126 (6,3%)	158 (7,9%)	206 (10,3%)	707 (35,4%)	802 (40,1%)	81 (5.9%)	118 (8.6%)	163 (11.9%)	485 (35.4%)	523 (38.2%)	6.1	18.2	15	25.3	35.4
Cereal and cereal products	1001 (61,2%)	176 (10,8%)	249 (15,2%)	164 (10%)	46 (2,8%)	794 (63.2%)	132 (10.5%)	174 (13.8%)	120 (9.5%)	37 (2.9%)	70.1	18	7.6	2.4	1.8
Confectionery	4 (0,2%)	31 (1,6%)	78 (4%)	609 (31,4%)	1216 (62,7%)	4 (0.3%)	20 (1.6%)	49 (4%)	373 (30.2%)	788 (63.9%)	0	0.7	0.4	20.7	78.2
Convenience foods	87 (15,2%)	161 (28,2%)	218 (38,2%)	97 (17%)	8 (1,4%)	53 (15.3%)	108 (31.2%)	133 (38.4%)	47 (13.6%)	5 (1.4%)	30.8	30.9	26.8	11.5	0
Dairy	254 (9,9%)	615 (23,9%)	605 (23,5%)	988 (38,3%)	116 (4,5%)	170 (10.9%)	439 (28.2%)	285 (18.3%)	595 (38.2%)	68 (4.4%)	21.8	61.6	7.3	8.8	0.4
Edible oils and emulsions	0 (0%)	0 (0%)	203 (41,3%)	211 (42,9%)	78 (15,9%)	0 (0%)	0 (0%)	160 (43.1%)	154 (41.5%)	57 (15.4%)	0	0	17.3	70.9	11.8
Eggs	18 (62,1%)	11 (37,9%)	0 (0%)	0 (0%)	0 (0%)	13 (61.9%)	8 (38.1%)	0 (0%)	0 (0%)	0 (0%)	72.1	27.9	0	0	0
Fish and fish products	74 (14,7%)	119 (23,7%)	138 (27,4%)	165 (32,8%)	7 (1,4%)	30 (8.4%)	81 (22.6%)	100 (27.9%)	142 (39.6%)	6 (1.7%)	9.7	19.8	24.8	45.1	0.6
Fruit and vegetables	727 (39,9%)	281 (15,4%)	572 (31,4%)	235 (12,9%)	7 (0,4%)	498 (39.1%)	191 (15%)	416 (32.7%)	165 (13%)	4 (0.3%)	66.3	14.6	14	5	0.1
Meat and meat products	111 (6,8%)	60 (3,6%)	124 (7,5%)	664 (40,4%)	685 (41,7%)	51 (7%)	26 (3.6%)	67 (9.3%)	335 (46.3%)	245 (33.8%)	3.4	2.3	10	66.7	17.7
Snack foods	8 (1,6%)	5 (1%)	48 (9,9%)	275 (56,7%)	149 (30,7%)	6 (1.8%)	3 (0.9%)	40 (12%)	185 (55.6%)	99 (29.7%)	1.8	0	9.2	54.1	34.8
Sauces and spreads	68 (7%)	120 (12,3%)	329 (33,8%)	281 (28,9%)	175 (18%)	56 (7.7%)	90 (12.4%)	230 (31.6%)	222 (30.5%)	129 (17.7%)	6.9	12.6	25.2	17.2	38.1
